# Erector spinae plane block with catheter for management of percutaneous nephrolithotomy: A three case report

**DOI:** 10.1097/MD.0000000000022477

**Published:** 2020-10-02

**Authors:** Andrew Resnick, Michael Chait, Steven Landau, Sandeep Krishnan

**Affiliations:** a Department of Anesthesiology, Wayne State University School of Medicine, Detroit; b Department of Anesthesiology, St. Joseph Mercy Oakland Hospital, Pontiac, MI, USA.

**Keywords:** erector spinae plane block, visceral pain, somatic pain, peri-operative pain management, case report

## Abstract

**Introduction:**

Percutaneous nephrolithotomy is a procedure used for management of refractory renal calculi. Oral and parenteral opioids, along with local anesthetic infiltration, neuraxial anesthesia, and paravertebral blocks are the most common methods of managing intra-operative and post-operative pain for these patients. The erector spinae plane block with catheter (ESPC) is a newer interfascial regional anesthetic technique that can be used to manage peri-operative pain in these patients.

**Clinical findings:**

Three patients complained of significant flank pain were scheduled for percutaneous nephrolithotomy under general anesthesia in the prone position.

**Diagnoses:**

Patients were diagnosed with large renal calculi.

**Therapeutic interventions:**

Patients received ESPC in the pre-operative holding area at the level of the T7 transverse process. The ESPCS were bolused with a solution of 30 mL 0.25% bupivacaine with 4 mg dexamethasone prior to surgery. Patients also received oral tramadol 50 mg and acetaminophen 1 g as part of the multimodal pain protocol prior to surgery. After the procedure, the patients were bolused with 0.25% bupivacaine or started on an infusion of 0.25% bupivacaine to manage their pain.

**Outcomes:**

No opioid or other pain medications, other than the local anesthetic solution given in the ESPCs, were used during the intra-operative or post-operative period for management of pain in these patients. Visual analogue scale (VAS) scores were below 4 for all patients in the post-operative period, and patients did not report any issues with post-operative nausea or vomiting.

**Conclusion:**

These patients were compared to 3 prior patients who had undergone percutaneous nephrolithotomy without ESPC. The 3 patients without ESPC placement reported increased VAS scores, had increased opioid/pain medication consumption intraoperatively and postoperatively, and had increased incidence of perioperative nausea when compared to our ESPC patients. Our report shows that ESPC, in combination with a multimodal pain protocol, can be a good option for management of patients undergoing percutaneous nephrolithotomy.

## 1 Introduction

Percutaneous nephrolithotomy (PCNL) is a minimally invasive surgical procedure often used for definitive treatment of refractory renal calculi. A nephrostomy tube is usually inserted after PCNL to provide adequate urinary drainage, hemostasis, and access for possible complications. However, nephrostomy tubes can cause significant postoperative pain and prolonged hospitalization which can lead to patient dissatisfaction.^[1]^

The main sources of acute postoperative pain after PCNL are visceral pain originating from the kidney and ureter, and somatic pain from the incision site. Visceral pain is primarily transmitted through the T10 to L2 spinal nerves and incisional pain is conducted via T8 to T12 due to the incision site often at the 10th to 11th intercostal space.^[2]^ Current modalities used to manage pain for patients undergoing PCNL include oral and parenteral opioid administration, local anesthetic infiltration, and certain neuraxial and regional anesthesia procedures including thoracic epidurals and paravertebral blocks.[^3^,^4^] These techniques have proven to provide appropriate analgesic coverage but are not without risks and potential side effects.^[5]^

In recent years, the erector spinae plane block (ESPB) has been gaining popularity as a regional acute pain technique for an assortment of procedures/pathologies.^[6]^ ESPB is an ultrasound-guided injection of local anesthetic into the paraspinal interfascial plane. The injectate is placed deep to the erector spinae muscle causing it to separate from the posterior surface of the transverse process.^[7]^ Benefits of using this technique include its lower risk profile and fewer contraindications when compared to neuraxial techniques.^[8]^ There has been significant recent effort to elucidate the exact mechanism of analgesic action for ESPB; early data has shown the mechanism of action to be local anesthetic action at the ventral and dorsal rami of the spinal nerves, but this is still being explored and not yet fully understood.[^9^,^10^,^11^] ESPB can be performed as a single shot injection or via catheter placement. Pain after single shot ESPB injections has been reported to recur after 2 to 3 h despite using long-acting local anesthetics.^[12]^ It has been hypothesized that the decreased duration of action for single shot injections is due to local anesthetic absorption. Thus, many practitioners are transitioning to the use of continuous catheters to provide longer lasting analgesia.^[12]^ In 2018, Kim et al published a single-patient case report of a patient who underwent placement of an erector spinae plane catheter (ESPC) for postoperative pain management after PCNL.^[13]^ Intraoperative opioid use or the incidence of postoperative nausea and vomiting (PONV) was not discussed. As the use of opioids intraoperatively have been shown to potentially lead to hyperalgesia and increased use of opioids postoperatively,[^14^,^15^] we sought to discover whether ESPC could be used to effectively manage intraoperative pain as well as postoperative pain while decreasing the side effects associated with opioid use.

In this case series, we report three cases of ESPC used for intraoperative and postoperative pain management of PCNL procedures. Each patient received an ESPC that helped achieve satisfactory analgesia while decreasing opioid consumption and PONV.

## 2 Case 1

### 2.1 Patient information

A 59-year-old female (97 kg, 160 cm) was scheduled for left PCNL due to a 9 mm stone in the left renal pelvis.

### 2.2 Therapeutic intervention

The ESPC was performed using ultrasound at the level of T7. The T7 transverse process was located using the inferior angle of the scapula as a landmark. A curvilinear 5-2 MHz ultrasound probe was placed on the patient's back in a cephalad-to-caudal fashion at the level of T7. The trapezius and erector spinae muscle were visualized on the left side, and a 19G Tuohy needle was advanced (in-plane to the ultrasound probe) in a caudal direction toward the left-sided transverse process of T7. After confirming that the tip of the needle was below the erector spinae muscle with a small bolus of our local anesthetic solution (30 mL of 0.25% bupivacaine with dexamethasone 4 mg), we injected 20 mL of the solution into the space, noting the caudal spread of the injectate on ultrasound. Then, a 20-gauge epidural catheter was inserted through the Tuohy needle and advanced 5 cm into the space. Placement of the catheter under the erector spinae muscle was confirmed by injecting the final 10 mL of the local anesthetic through the catheter while visualizing the spread of the solution on ultrasound. The catheter was then secured using the LOCKIT Plus catheter securement device, which allows for mechanical securement of the catheter while maintaining its patency. Additionally, it provides easy access for visual inspection of the insertion site via a transparent window.

### 2.3 Follow-up and outcomes

The PCNL was performed in the prone position using general anesthesia. The patient received acetaminophen 1 g in the preoperative area as part of a multimodal pain protocol (she refused the tramadol 50 mg which is also part of multimodal pain protocol). She underwent general anesthesia and received no opioid or other pain medications during the 118-min procedure; she was extubated without complication at the end of the case. Pain scores in the Post-Anesthesia Care Unit (PACU) were assessed by nursing using visual analogue scale (VAS) scores and recorded in the medication administration record (MAR). All medications given for pain and PONV were recorded in the MAR as well.

The patient remained in the PACU for 231 min and reported VAS scores of 0/0/0/0/0. She received ondansetron 4 mg intraoperatively and 4 mg prophylactically in PACU due to a patient-reported history of severe PONV. The patient was given an additional 10 mL bolus of 0.25% bupivacaine 1-h prior to discharge, and the catheter was removed with the tip intact. The patient was discharged home from the PACU without complications and reported no pain at all.

### 2.4 Patient perspective

The patient returned to the hospital for a left-sided ureteral stent exchange 15 days later and reported that she had no residual pain in the days following her previous procedure.

## 3 Case 2

### 3.1 Patient information

A 66-year-old female (66 kg, 160 cm) presented for left PCNL due to a 1 cm left renal stone.

### 3.2 Therapeutic intervention

In the preoperative holding area, the patient underwent ESPC with catheter placement at T7 using a technique identical to Case 1. She was given 30 mL of the 0.25% bupivacaine and dexamethasone 4 mg solution.

### 3.3 Follow-up and outcomes

The patient received acetaminophen 1 g and tramadol 50 mg prior to surgery as part of the multimodal pain protocol. She underwent general anesthesia for her 85-min procedure; intraoperatively, the patient received no opioid or other pain medications. She received ondansetron 4 mg prophylactically to prevent PONV.

Upon arrival in PACU, the patient admitted to having mild pain and reported a VAS score of 4. The ESPC was bolused with 5 mL of 0.25% bupivacaine, after which she reported VAS scores of 0/0. The patient was admitted overnight for observation due to concern for bleeding at the surgical site. The ESPC was connected to a continuous infusion in PACU; the settings for the infusion were 6 mL an hour of 0.25% bupivacaine with a patient-controlled bolus of 4 mL every 20 min as needed. The patient did not use the patient-controlled bolus function and reported having minimal pain overnight. She reported VAS scores of 0/0/0/1/0. She was discharged the following afternoon without complications after removal of the catheter with the tip intact.

## 4 Case 3

### 4.1 Patient information

A 71-year-old female (132 kg, 157 cm) underwent right PCNL for a 7 mm renal stone.

### 4.2 Therapeutic intervention

As in Case 1 and Case 2, the patient underwent preoperative placement of ESPC. She was given 30 mL of the local anesthetic solution prior to surgery as in Case 1 and Case 2.

### 4.3 Follow-up and outcomes

Patient received acetaminophen 1 g and tramadol 50 mg as part of the multimodal pain protocol. The 116-min procedure was performed under general anesthesia; she received no intraoperative opioid or other pain medication. She did receive a prophylactic dose of ondansetron 4 mg to prevent PONV.

In the PACU, the patient reported VAS scores of 0/0/0/0/0/0/0 and denied PONV. One-hour prior to discharge, the ESPC was bolused with 10 mL of 0.25% bupivacaine and the catheter was removed with the tip intact. She was discharged after a 237-min stay in PACU without pain.

## 5 Discussion

Neuraxial and regional blockade have commonly been used for relieving pain from a myriad of surgical procedures and pain syndromes. ESPC placement has emerged as an effective regional technique, providing excellent analgesia with reduced opioid requirements.[^16^,^17^] Additionally, these blocks are relatively simple to perform with a favorable safety profile. They can be used for a variety of procedures in which multiple dermatomes are targeted for the purpose of providing adequate sensory blockade. Jain et al reported excellent sensory blockade using this regional technique for breast, abdominal, and thoracic procedures, as well as for patients suffering from chronic neuropathic pain.^[18]^ ESPC mitigates potential procedural complications including hypotension from epidural analgesia and vascular puncture from paravertebral block. Additionally, the risk of pneumothorax associated with intercostal nerve block and interpleural block is significantly reduced.^[19]^ Its continuous nature, extensive craniocaudal spread, distance from the surgical field, and excellent sensory blockade could make ESPC superior to many of our current first line analgesic treatments.

For patients undergoing PCNL procedures, much of the pain produced is visceral in nature secondary to the large size of the renal calculus with subsequent stretching of capsular fibers involving the T10 to L2 dermatomes.^[13]^ The local anesthetic administered during the ESPC has a wide area of distribution and likely spreads via action on the ventral and dorsal roots affecting various levels from T1 to T11, based on various studies.[^20^,^21^,^22^] Hence, patients undergoing PCNL have the potential to greatly benefit from this type of peripheral nerve block.

In order to further assess the potential benefit of the ESPC, we retrospectively examined the medical records of the three previous patients at our institution who underwent PCNL without ESPC. The first patient we examined received acetaminophen 1 g and tramadol 50 mg as part of the preoperative multimodal pain protocol. She received fentanyl 200 mcg and ketamine 100 mg intraoperatively, and, in PACU, she received hydromorphone 0.5 mg IV and reported VAS scores of 5/0/0/0. She also required ondansetron 4 mg and haloperidol 1 mg for PONV in PACU. The second patient received acetaminophen 1 g and tramadol 50 mg preoperatively, fentanyl 200 mcg intraoperatively, and ketorolac 30 mg and one hydrocodone/acetaminophen 5 mg/325 mg pill in PACU. Her VAS scores in PACU were 3/3/2/2. The third patient received acetaminophen 1 g and tramadol 50 mg preoperatively, fentanyl 100 mcg intraoperatively, and two hydromorphone 0.5 mg IV pushes in PACU. She also required two hydrocodone/acetaminophen 10 mg/325 mg pills for pain during her 1-day admission as well as ondansetron 4 mg on two separate occasions for PONV. Her VAS scores in PACU and on the medical floor were 5/6/2/0/0/6/3/6/3/7/3/3. The patients we examined without ESPC placement had increased VAS scores post-operatively, increased opioid/pain medication consumption intraoperatively and postoperatively, and increased incidence of perioperative nausea when compared to our ESPC patients (see Tables 1 and 2).

**Table 1:**
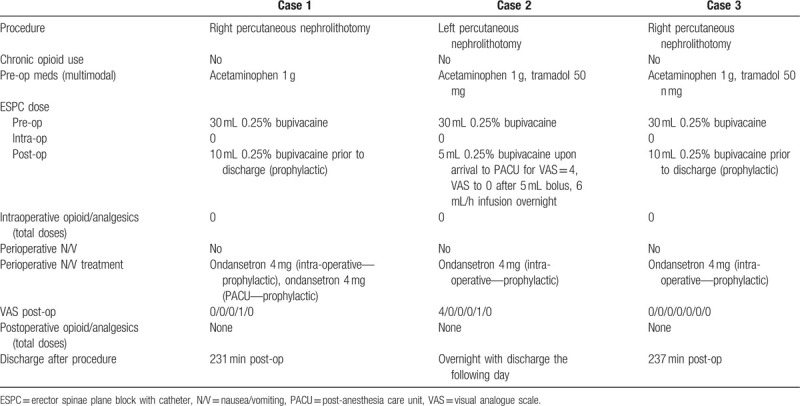
Patients receiving ESPC prior to surgery.

**Table 2:**
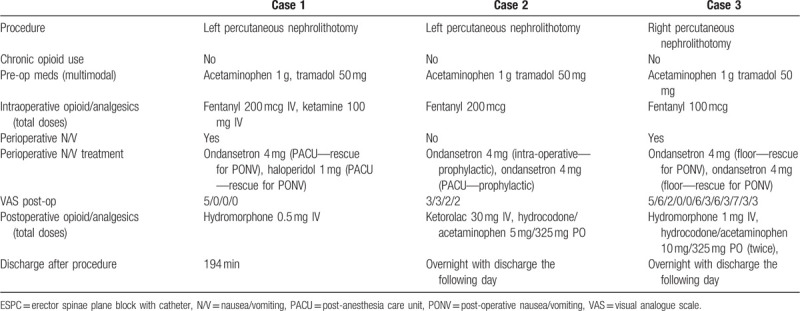
Patients not receiving ESPC for surgery.

In our experience of patients who underwent ESPC for PCNL at our institution, intraoperative and postoperative opiate usage, PONV requiring treatment, and VAS scores were all effectively decreased. Our case report shows that ESPC for PCNL can be an effective method to manage intraoperative and postoperative pain in combination with a multimodal pain protocol.

## Author contributions

**Conceptualization:** Sandeep Krishnan.

**Data curation:** Sandeep Krishnan.

**Formal analysis:** Sandeep Krishnan.

**Investigation:** Andrew Resnick, Michael Chait, Sandeep Krishnan.

**Methodology:** Sandeep Krishnan.

**Project administration:** Sandeep Krishnan.

**Supervision:** Sandeep Krishnan.

**Writing – original draft:** Andrew Resnick, Michael Chait, Sandeep Krishnan.

**Writing – review & editing:** Andrew Resnick, Michael Chait, Steven Landau, Sandeep Krishnan.
